# Metabolism and Cancer: Old and New Players

**DOI:** 10.1155/2013/293201

**Published:** 2013-06-23

**Authors:** Claudia Cerella, Carine Michiels, Roderick H. Dashwood, Young-Joon Surh, Marc Diederich

**Affiliations:** ^1^Laboratoire de Biologie Moléculaire et Cellulaire du Cancer (LBMCC), Hôpital Kirchberg, 9 rue Edward Steichen, L-2540 Luxembourg, Luxembourg; ^2^Laboratory of Biochemistry and Cellular Biology (URBC), NAmur Research Institute for LIfe Science (NARILIS), University of Namur, 5000 Namur, Belgium; ^3^Cancer Chemoprotection Program, Linus Pauling Institute, 307 Linus Pauling Science Center, Oregon State University, Corvallis, OR 97331, USA; ^4^Tumor Microenvironment Global Core Research Center, College of Pharmacy, Seoul National University, Seoul 151-742, Republic of Korea

Altered metabolism represents one of the oldest hallmarks associated with cancer. The aberrant metabolic profile deals primarily with the well-known switch of transformed cells towards aerobic glycolysis from mitochondrial oxidative phosphorylation. In addition, it deals with the exacerbation of several biosynthetic pathways interconnected with the increased glycolytic flux, eventually conferring selective metabolic advantages.

The distinctive and ubiquitous nature of this altered metabolic profile expressed by cancer cells from various origins or tissues turns the modulation/reversion of these aberrations as an amenable strategy for new anticancer therapies. Despite Otto Warburg's pioneering observations, targeting cancer cell metabolism for therapeutic purposes still remains theoretical. Several factors have contributed to this situation. Primarily, for many years, cancer has been treated as a homogeneous and too simplistic cellular system, where the impairment of proliferation/viability of the most abundant differentiated cancer cells has been extensively pursued. This approach, however, has not been successful in preventing tumour relapse. Cancer is nowadays considered a complex network, including cancer cells at different stages of differentiation, as well as noncancer cells from the tumour microenvironment. These cells exert specific functions further sustaining cancer progression, by maintaining a proinflammatory environment, by inducing activation of angiogenesis and by evading immune responses. The cellular heterogeneity implies more subtle forms of mutual interaction and communication among different cells and with the microenvironment, which may be the niche for future innovative anticancer therapeutic interventions.

In this renewed view of cancer etiology, unexpected crosstalk is emerging between metabolic mediators/processes and pathological alterations.

This special issue focuses on “Metabolism and cancer: old and new players” and intends to stimulate the discussion about new emerging targetable mediators of cancer metabolism and to evaluate possible noncanonical roles of old players in tumorigenesis as well as in cancer metabolism.

The impairment of cancer cell viability by induction of cell death remains the final outcome of many anticancer strategies. Besides apoptosis, other modes of cell death have been defined and are attracting interest as alternative therapeutically exploitable approaches to impact apoptosis-resistant forms of cancer. The review by S. Fulda, “*Alternative cell death pathways and cell metabolism*,” draws attention to the programmed form of necrosis, namely, necroptosis, and discusses the relevance of metabolic pathways involved in its modulation. The author suggests a possible interconnection between alterations of redox signaling pathways mediating necroptosis and mitochondrial impairment, which implies the latter as a crucial regulator of redox imbalance. 

An ideal anticancer therapy should be able to efficiently target cancer stem cells. This requires the discovery of markers selectively identifying malignant stem cells. The review by C. Pecqueur et al., “*Targeting metabolism to induce cell death in cancer cells and cancer stem cells*,” addresses the relationship between cancer cell metabolism and evasion from apoptosis by comparing the main features of cancer cells and cancer stem cells. The authors correlate the peculiar metabolic characteristics of these two types of cancer cells with their ability to evade apoptosis.

Over the last 10 years, noncanonical roles for many factors implicated in apoptosis were published. The family of B-cell lymphoma-2 (Bcl-2) proteins represents one of the most interesting examples, with several members exhibiting essentially pro- and apoptotic functions. The paper by J. Michels et al., “*Functions of Bcl-xL at the interface between cell death and metabolism*,” reviews new and intriguing properties of the Bcl-2 homolog Bcl-xL (B-cell lymphoma extra-large) linked to regulation of bioenergetic metabolism, which in turn controls important processes including mitochondrial ATP synthesis, Ca^2+^ flux, autophagy, mitosis, and protein acetylation.

Cancer cells rely on glycolysis rather than on oxidative phosphorylation to fulfill their energy needs. In conditions of glucose deprivation, however, cancer cells may reactivate mitochondrial bioenergetics as part of a prosurvival strategy. The study by R. Palorini et al., “*Mitochondrial complex I inhibitors and forced oxidative phosphorylation synergize in inducing cancer cell death,”* shows that mitochondrial complex I inhibitors sensitize cancer cells to cell death under glucose depletion. Interestingly, combined treatments affecting glycolysis and leading to mitochondria impairment are ineffective on immortalized cells or cancer cells cultivated under high glucose conditions. Their results suggest that the forced switch from glycolysis to oxidative phosphorylation combined with the use of mitochondrial inhibitors may be used as alternative therapeutic approach to increase the sensitivity of cancer cells to death.

Identification of specific metabolic intermediates as targets for future anticancer treatments may be at the basis for attempting a reversal of tumor metabolism to normal conditions, with the rationale of impairing cancer growth. Pyruvate kinase M2 (PKM2) catalyzing the final rate-limiting reaction of glycolysis currently attracts much interest because of its multiple emerging intracellular functions. The review by N. Wong et al., “*PKM2, a central point of regulation in cancer metabolism,*” gives an overview of our current knowledge about this important enzyme, discussing several of its potential contributions to tumorigenesis.

A limiting factor in exploiting cancer metabolism for therapeutic purposes is the lack of availability of agents acting as specific modulators of cancer metabolism. The review by C. Cerella et al., “*Natural compounds as regulators of the cancer cell metabolism,”* covers this aspect, focusing on natural compounds as untapped potential regulators of cancer cell metabolism. The authors discuss some of the most important pathways and factors implicated in altered cancer metabolism and give an overview of compounds extracted from natural sources potentially targeting these different aberrantly regulated metabolic key intermediates.

The link between obesity, insulin resistance, and cancer is explored from different sides in three papers of this special issue, interestingly dealing all with hormonal forms of cancer. The paper by C. Brosseau et al., “*Role of insulin-like growth factor binding protein-3 in 1, 25-dihydroxyvitamin-D*_3_ *-induced breast cancer cell apoptosis,”* aims at identifying the mechanisms involved in 1, 25-dihydroxyvitamin D_3_-induced apoptosis in breast cancer cells. Their strategy suggests a role for insulin-like growth factor binding protein-3 (IGFBP-3) in vitamin D-induced apoptotic signaling and the involvement of impaired secretion of IGFBP-3 in acquired chemoresistance. 

The review by J. S. Byun and K. Gardner, “*C-terminal binding protein: a molecular link between metabolic imbalance and epigenetic regulation in breast cancer*,” introduces the reader to the concept of metabolic transduction by stressing the link between shifts in carbohydrate metabolism and alterations in epigenetic regulatory mechanisms. In particular, these authors investigate the family of transcriptional repressors called C-terminal binding proteins (CtBPs), whose function is controlled by NAD^+^ levels, as an example of factors interacting and modulating the activity of different histone deacetylases (HDACs) depending on the metabolic status.

Finally, the review by J. H. Gunter et al., “*New players for advanced prostate cancer and the rationalisation of insulin-sensitising medication,*” points to the roles played by metabolic disorders (especially related to insulin resistance) in favoring anticancer treatment failure and consequent higher cancer-specific mortality. The authors discuss the promising use of insulin-sensitizing drugs as potential anticancer agents to be used in combinational therapies. 

Although alterations in cellular metabolism are an old acquaintance in cancer etiology, we still need to do more before we can translate our knowledge into new and specific anticancer therapies. This final outcome will mostly depend on an in-depth identification of the mutual modulation between metabolism processes and factors that regulate proliferation and death/survival of cancer cells. With this special issue, we hope to offer new and relevant insights towards a critical reevaluation of this rapidly moving field.

*Claudia Cerella* *Claudia Cerella* *Carine Michiels* *Carine Michiels* *Roderick H. Dashwood* *Roderick H. Dashwood* *Young-Joon Surh* *Young-Joon Surh* *Marc Diederich* *Marc Diederich*

